# Estrogen receptor alpha gene polymorphisms and risk of HBV-related acute liver failure in the Chinese population

**DOI:** 10.1186/1471-2350-13-49

**Published:** 2012-06-24

**Authors:** Zehui Yan, Wenting Tan, Yunjie Dan, Wenli Zhao, Chunqing Deng, Yuming Wang, Guohong Deng

**Affiliations:** 1 Institute of Infectious Diseases, Southwest Hospital, Third Military Medical University, Chongqing, 400038, P. R. China; 2 Institute of Immunology, Third Military Medical University, Chongqing, 400038, P. R. China

## Abstract

**Background:**

The sexual dimorphism of hepatitis B virus (HBV) -related liver diseases is related with estrogen and its receptors. Recent reports indicate that abnormal expression of estrogen receptor alpha (ESR1) may be a hallmark for the progression of liver disease and HBV carriers presenting variant ESR1 have an extremely aggressive clinical course. Here we examine whether the *ESR1* polymorphisms or its haplotypes are related to HBV-related acute liver failure (ALF) risk among chronic HBV carriers in a Chinese population.

**Methods:**

A total of 1216 unrelated Han Chinese HBV carriers were recruited in this hospital-based case–control study, including 359 HBV surface antigen (HBsAg) carriers affected with ALF and 857 asymptomatic HBsAg carriers. Two *ESR1* haplotype tagging polymorphisms, c.30 T > C (rs2077647) and c.453-397 T > C (rs2234693), were genotyped by polymerase chain reaction-restriction fragment length polymorphism (PCR-RFLP) assay.

**Results:**

We observed a significantly increased susceptibility to HBV-ALF associated with the c.30 C allele (P = 8.65 × 10^-4^), c.453-397 C allele (5.37 × 10^-4^) and [c.30 C; c.453-397 C] haplotype (Dominant model, *P* =0.0004, odds ratio = 1.53, 95% CI 1.23 ~ 1.96) compared with the T alleles and [c.30 T; c.453-397 T] haplotype of c.30 T > C and c.453-397 T > C polymorphisms, respectively.

**Conclusions:**

Our study suggests that [c.30 C; c.453-397 C] hapotype may be a risk factor for genetic susceptibility to HBV-related ALF in the Chinese population. It also emphasizes the importance of ESR1 in the pathophysiology of HBV-related ALF on the population level.

## Background

Acute liver failure (ALF) is a condition in which rapid deterioration of liver function results in altered mentation and coagulopathy in previously normal individuals. The most widely accepted definition of ALF includes evidence of coagulation abnormality, usually an INR ≥ 1.5, and any degree of mental alteration (encephalopathy) in a patient without preexisting cirrhosis and with an illness of <26 weeks duration [1]. ALF is the final common pathway of severe hepatocyte injury. The etiology of ALF shows worldwide variation, prominent causes include drug-induced liver injury, viral hepatitis, autoimmune liver disease and shock or hypoperfusion. Unlike western countries, ALF is one of the end-staged liver diseases caused mainly by hepatitis B virus (HBV) infection (at least 60%) in China [2]. However, regardless of etiology, chronic hepatitis B progresses at unequal rates between males and females, being more frequent in men than in women [3]. This sexual dimorphism and the greater progression to the end-staged liver diseases in men and postmenopausal women may be due, at least in part, to lower production of estrogen and a reduced response to the action of estrogen [4].

Estrogen is a potent endogenous antioxidant which attenuates induction of redox sensitive transcription factors and hepatocyte apoptosis by inhibiting generation of reactive oxygen species [5]. The effects of estrogens are mediated by estrogen receptors (ESRs). When estrogen binds to its receptor, it would activate and regulate multiplegenes, such as activator protein-1, B-cell lymphoma 2, nuclear factor κB, tumor necrosis factor α and interleukin-6 [6-8]. There are two known ESRs: estrogen receptor α (ESR1) and estrogen receptor β (ESR2). The majority of the biological effects of estrogen are mediated by ESR1 in the liver [9]. ESR1 has been well characterized in human liver [10], hepatic cytosolic ESR1 content was detected on the progression of chronic liver disease [11-13]. The abnormal ESR1 expressions in the liver have been implicated in stimulating hepatocyte injury and may act as liver disease inducers or promoters [14]. Furthermore, variant ESR1 is expressed to a greater extent in male patients than in females, even at an early stage of chronic liver disease [15,16]. Therefore, variant ESR1 may be a hallmark for the progression of liver disease and HBV carriers presenting variant ESRa1 have an extremely aggressive clinical course [17].

Investigators have also hypothesized the *ESR1* (NM_000125.3) genetic variants may influence an individual's estrogen-sensitive phenotypes [18]. We have screened single nucleotide polymorphisms (SNPs) systematically in the *ESR1* gene and two linkage disequilibrium (LD) blocks covering the *ESR1* gene were identified [19]. Two polymorphisms, *ESR1* c.30 T > C (rs2077647, previously reported T29C, exon 1) and c.453-397 T > C (rs2234693, previously reported IVS1 T-401 C, intron 1), which were identified as haplotype tagging SNPs, influence the susceptibility to persistent HBV infection [19], HBV-related liver cirrhosis [20] and hepatocellular carcinoma [21]. We also observed that the relative mRNA levels of the risk c.30 C allele were consistently higher than those of the c.30 T allele in c.30 T > C heterozygotes [21]. Functional analyses also showed that the c.453-397 T > C polymorphism is a novel c.453-397 C allele-specific and c-myb-dependent enhancer-like cis-acting regulatory variation and could be part of the genetic variations underlying the susceptibility of individuals to HBV-related diseases [20].

Host genetic polymorphisms may involve the the pathophysiology of HBV-ALF. We have reported that the -592 C allele and the -1082A-819 C-592 C haplotype in the *IL-10* gene promoter were associated with an increased susceptibility to ALF in HBV carriers [22]. In this study, we hypotheses that *ESR1* may be another appropriated biological candidate susceptibility gene for HBV-ALF. It is expected that the *ESR1* genetic variation could influence the effects of estrogens, which in turn results in genotype-dependent differences in risk of HBV-ALF. Therefore, we selected the two haplotype tagging SNPs (c.30 T > C and c.453-397 T > C) from previously association LD block of *ESR1* and examined their relationships with the susceptibility to HBV-ALF in a large sample of case–control cohort.

## Methods

### Study participants

Hospital-based case–control study was conducted at Southwest Hospital (Chongqing, China) between February 2001 and December 2009. A total of 1216 unrelated Han Chinese HBV carriers were recruited, including 359 HBV surface antigen (HBsAg) carriers affected with ALF and 857 asymptomatic HBsAg carriers. All HBsAg carriers were positive for both HBsAg and antibody to HBV core antigen (anti-HBc) of the IgG type for at least 12 months. All carriers were measured with liver function tests, serum immunologic marker screening, at least one liver image examination (Ultrasonography, Computed Tomography, Magnetic Resonance Imaging and Fibroscan), and 113 (9.3%) were examined with histological biopsy. All carriers had no serologic evidence for coinfection or superinfection with hepatitis A virus, hepatitis C virus, hepatitis D virus, hepatitis E virus, and human immunodeficiency virus.

ALF was defined as liver failure with jaundice, coagulation abnormality (usually an INR ≥1.5), and any degree of mental alteration (encephalopathy) in a patient with an illness of less than 26 weeks duration. All patients with ALF had no evidence with preexisting cirrhosis. Information on acetaminophen and idiosyncratic drug and treatment history of these patients was obtained mainly from clinical records and short telephone interviews when necessary. The carriers, who had history of acetaminophen overdose and idiosyncratic drug reactions, were excluded from the HBV-ALF group. Asymptomatic HBV carriers were diagnosed according to the following criteria: (1) lack of any clinical symptoms; (2) normal liver enzyme tests; (3) normal peripheral blood leucocyte (4 ~ 10 × 10^9^/L) and platelet (100 ~ 400 × 10^9^/L) counts; (4) serum albumin >39 g/L, globulin <35 g/L, and the ratio of albumin to globulin (A/G) >1.5; (5) normal prothrombin time and serum total bilirubin; (6) no abnormal findings on abdominal ultrasound scans; (7) no esophageal varix revealed by electronic gastroscopy. The examininations or tests were performed once every 6 months from February 2001 to December 2007. The carriers, who occurred to the hepatitis symptoms or cirrhosis evidences during the follow-up, were also excluded from the ASC group.

Further clinical and demographic characteristics of the studied population are shown in Table 1. All subjects provided informed consent to participate in the study, as approved by the ethical committee of Southwest Hospital, Chongqing, China.

**Table 1:** **Distribution of Selected Characteristics and*ESR1*Polymorphisms in Participants**

Characteristic	ASC	HBV-ALF	P value
	(*n* =857)	(*n* = 359)	
Gender, no. (%)			
Men	510 (59.5)	298 (83.0)	
Women	347 (40.5)	61(17.0)	<0.001
Age (years), mean (SD)	36.6 ± 11.3	41.1 ± 12.7	<0.001
Alcohol drinkers, no. (%)	160(18.7)	121 (33.7)	<0.001
HBeAg positive, no (%)	298(34.8)	107(29.8)	0.094
TBil (μmol/L)	13.4 ± 4.3	321.1 ± 166.1	
ALT (IU/L)	27.9 ± 10.7	410.1 ± 541.6	
c.453-397 T > C (rs2234693)			
TT, no. (%)	330(38.4)	109(30.4)	
TC, no. (%)	424(49.6)	182(50.7)	
CC, no. (%)	103(12.0)	68(18.9)	
T allele, no. (%)	1086(62.4)	400 (55.7)	
C allele, no. (%)	632(36.9)	318 (44.3)	5.37 × 10^-4^
c.30 T > C (rs2077647)			
TT, no. (%)	418(48.8)	143 (39.8)	
TC, no. (%)	348(40.6)	160 (44.7)	
CC, no. (%)	91 (10.6)	56 (15.6)	
T allele, no. (%)	1184(69.1)	446 (62.1)	
C allele, no. (%)	530 (30.9)	272 (37.9)	8.65 × 10^-4^

**Notes:** “Drinker” was defined as alcohol consumption of ≥40 g/week for men and ≥20 g/week for women, which included occasional drinkers and daily drinkers. The genotype distributions of SNPs in each group were in Hardy–Weinberg equilibrium. *P* values were given for the comparison between ASC and HBV-ALF groups by *χ*^2^ tests. TBil, total bilirubin; *ASC*, asymptomatic HBV carriers; *HBV-ALF*, patients with HBV-related acute liver failure.

### DNA extraction and genotyping

Genomic DNA was extracted from peripheral blood leukocytes from 5 mL whole blood by using standard phenol/chloroform protocols. DNA samples were diluted to10 ng/μL and distributed into 96-well plates (DNA panels), with 94 samples and 2 controls (DNA-free water) in each plate. Entrez nucleotide database (http//ncbi.nlm.nih.gov) NT_025741.15:g and dbSNP ID were used as the reference sequences for *ESR1* genomic and cDNA sequences. DNA sequence variations are named according to the nomenclature recommendations from http://www.hgvs.org/mutnomen/recs-DNA.html[23,24]. The *ESR1* c.30 T > C and c.453-397 T > C polymorphisms were genotyped by polymerase chain reaction-restriction fragment length polymorphism (PCR-RFLP) analysis in our case–control population.

For the c.30 T > C polymorphism, an amplification using the forward primer 5’-GACCATGACCCTCCACACCAAAGGATC -3(G, mismatch base) and reverse primer 5’- ACCGTAGACCTGCGCGTTG -3’ was performed at an annealing temperature of 61 °C. The reaction yielded a 220 bp amplicon. A BamH I recognition site was introduced by 1-base mismatch (underlined base) in the forward primer. Three microliters PCR products were digested with 4U BamH I (Toyobo, Japan). Digested amplicons from the homozygotes for c.30 C allele appeared as 197-bp and 23-bp bands on the agarose gel electrophoresis, while homozygotes for c.30 T allele appeared as a 220-bp band. Heterozygotes of c.30TC had all 3 of these bands.

For the c.453-397 T > C polymorphism, amplification using the forward primer 5’-CATGAACCACCATGCTCAGTC-3’ and reverse primer 5’-ACTCTCTGGGAGATGCAGCAG-3’ was performed at an annealing temperature of 63 °C. The reaction yielded a 548 bp amplicon. Three microliters PCR products were digested with 4U PvuII (Toyobo, Japan), and separated on 3% agarose gel and stained with ethidium bromide for visualization under UV light. Digested amplicons from the homozygotes for c.453-397 T allele appeared as 346-bp and 202-bp bands on the agarose gel electrophoresis, while homozygotes for c.453-397 C allele appeared as a 548-bp band. Heterozygotes of c.453-397TC had all 3 of these bands.

Genotyping was performed in a blind manner without information of subjects’ case/control status. The accuracy of genotyping data for SNP obtained from PCR-RFLP was validated by direct sequencing of 90 masked, random samples of patients.

### Haplotype construction and statistical analysis

Allele frequencies for each SNP were determined by gene counting, and the significance of deviations from Hardy-Weinberg equilibrium was tested using the random-permutation procedure implemented in the Arlequin package (http://lgb.unige.ch/arlequin/). *ESR1* haplotypes were assigned by the PHASE program [25]. Pair-wise linkage disequilibrium (LD) between SNPs was analyzed by LDA [26].

Statistical analysis was performed using SPSS software (version 9.0; SPSS Inc, Chicago, IL). A p-value less than 0.05 was considered significant. *χ*^2^ tests were performed to examine the differences in the allele frequency and genotype distribution between groups. Multivariable logistic regression analysis was performed to adjust risk factors such as age, sex, and alcohol use. The association between genotyped polymorphisms and the risk of disease was estimated by *P* values, odds ratios (ORs), and 95% confidence intervals (95% CIs).

## Results

The selected characteristics and genotype distributions of the *ESR1* c.30 T > C and c.453-397 T > C polymorphisms in ASCs and HBV-ALF groups are summarized in Table 1. Although efforts were made to obtain good matches on age and sex between ASCs and HBV-ALF groups, HBV-ALF groups were older and consisted more men (P < 0.001). The difference in the alcohol consumption status between the ASCs and HBV-ALF groups (P < 0.001) was observed,which may due to gender difference since few women drink in China. There was no significant difference with the percentage of HBeAg positive (P =0.094) between the asymptomatic HBV carriers (34.8%) and the patients of HBV-related ALF (29.8%). The genotype distributions for the two SNPs were in Hardy–Weinberg equilibrium in each group. The c.30 T > C and c.453-397 T > C polymorphisms were in linkage disequilibrium with each other (D' = 0.796, r^2^ = 0.538, Q value = 0.951). The c.30 C and c.453-39 C allele frequencies were significantly higher in HBV-ALF patients than those in ASCs (*P* < 0.05). Subjects bearing at least one c.453-39 C and c.30 C alleles had an increased susceptibility to HBV-related ALF compared with those without c.453-39 C and c.30 C alleles (c.453-397 T > C, *P =* 5.37 × 10^-4^; T29C, *P =*8.65 × 10^-4^) in our case–control sample.

To decrease the bias of age and sex on the effect estimates, we conducted stratification analysis for age and sex in total 1216 case and control individuals (Table 2). The association between two haplotype tagging SNPs (c.30 T > C and c.453-397 T > C) and HBV-ALF remained significant in male patients (c.453-397 T > C, *P* =0.005; c.30 T > C, *P* = 0.022), female patients(c.453-397 T > C, *P* = 0.033; c.30 T > C, *P* = 0.021), patients with age ≥ 40 years (c.453-397 T > C, *P* =0.005; c.30 T > C, *P* = 0.0003), and patients with age < 40 years (c.453-397 T > C, *P* = 0.011; c.30 T > C, *P* =0.003). As Table 2 showed, the logistic regression analysis with adjustment for covariates, including age, sex, HBeAg status and alcohol consumption also suggested that the genotype effects of these two SNPs were significantly associated with the risk to HBV-ALF in both gender and patients at any age (with an age ≥ 40 years or < 40 years).

**Table 2:** **Stratification Analysis of Age and Gender on Association between*ESR1*c.30 T > C & c.453-397 T > C Genotypes and Different Groups**

	c.453-397 T > C				c.30 T > C			
	AsC	HBV-ALF	P value	OR (95%CI)	AsC	HBV-ALF	P value	OR (95%CI)
Men only.	n = 510	n = 298			n = 510	n = 298		
TT	193(37.8)	94(31.5)			233(457)	121(40.6)		
TC	263(51.6)	149(50.0)			230(45.1)	131(43.9)		
CC	54(10.6)	55(18.5)	0.003	1.39(1.12-1.73)	47(9.2)	46(15.4)	0.026	1.47(1.06-2.17)
T allele	649(63.6)	337(56.5)			696(68.2)	373(62.6)		
C allele	371(36.4)	259(43.5)	0.005		324(31.8)	223(37.4)	0.022	
Women only	n = 347	n = 61			n = 347	n = 61		
TT	137(39.5)	15(24.6)			185(53.3)	22(36.1)		
TC	161(46.4)	33(54.1)			118(34.0)	29(47.5)		
CC	49(14.1)	13(21.3)	0.022	1.56(1.08-2.35)	44(12.7)	10(16.4)	0.037	1.63(1.07-2.47)
T allele	435(62.7)	63(51.6)			488(70.3)	73(59.8)		
C allele	259(37.3)	59(48.4)	0.021		206(29.7)	49(40.2)	0.021	
Age ≥ 40 y	n = 277	n = 166			n = 277	n = 166		
TT	118(42.6)	54(32.5)			151(54.5)	71(42.8)		
TC	135(48.7)	83(50.0)			101(36.5)	74(44.6)		
CC	24(8.7)	29(17.5)	0.008	1.83(1.21-2.63)	25(9.02)	21(12.7)	0.041	1.62(1.08-2.54)
T allele	371(67.0)	191(57.5)			403(72.7)	216(65.1)		
C allele	183(33.0)	141(42.5)	0.005		151(27.3)	116(34.9)	0.0003	
Age < 40 y	n = 580	n = 193			n = 580	n = 193		
TT	212(36.6)	55(28.5)			267(46.0)	72(37.3)		
TC	289(49.8)	99(51.3)			247(42.6)	86(44.6)		
CC	79(13.6)	39(20.2)	0.036	1.35(1.09-2.21)	66(11.4)	35(18.1)	0.024	1.54(1.07-1.98)
T allele	713(61.5)	209(54.1)			781(68.2)	230(59.6)		
C allele	447(38.5)	177(45.9)	0.011		369(31.8)	156(40.4)	0.003	

**Notes:***P* values were given for the comparison of the allele effect between ASC and HBV-ALF groups by *χ*^2^ tests. Odds ratios (OR) and their 95% confidence interval (95%CI) were given for the comparison of the genotype effect between ASC and HBV-ALF groups by logistic regression analysis with adjustment for covariates, including age, sex, HBeAg status and alcohol consumption. *ASC*, asymptomatic HBV carriers; *HBV*- *ALF*, patients with HBV-related acute liver failure.

Haplotypes based on the c.30 T > C and c.453-397 T > C polymorphisms were constructed to derive haplotypes specifically correlated with HBV-ALF. Four haplotypes, [T; T], [T; C], [C; T], and [C; C], were observed, and two haplotypes, [T; T] and [C; C], had frequencies more than 10%. The estimated haplotype distribution differed significantly between the ASCs and HBV-ALF groups (*χ*^2^ =19.74, *P* =0.0002, Table 3). The frequency of [T; T] haplotype decreased significantly in the HBV-ALF group, as compared to ASCs. We categorized the *ESR1* haplotypes into three groups, i.e., ‘[T; T]/[T; T]’ homozygotes, ‘[T; T]]/[-,-]’ heterozygotes, and ‘[-;-]/[-;-]’ homozygotes (‘[-;-]’means non-[T; T] haplotypes, Table 3). Logistic regression analysis with adjustment for age, sex, HBeAg status and alcohol consumption indicated significant differences in the distribution of the three haplotype groups between the ASC and HBV-ALF groups. Non-[T; T]](‘[-;-]/[-;-]’) haplotypes had a significantly increased risk to HBV-ALF (Dominant model, *P* = 0.0004, odds ratio = 1.53, 95% CI 1.23 ~ 1.96).

**Table 3:** **Results of the Association Test for*ESR1*Haplotypes between ASC and HBV-related ALF Groups**

Characteristic	AsC	HBV-ALF	*P* value	OR (95%CI)
	(2n = 1714)	(2n = 690)		
Haplotype, no. (%)				
[T; T]	1023(59.7)	365(50.8)		
[T; C]	142(8.3)	82(11.5)		
[C; T]	54(3.1)	36(5.0)		
[C; C]	495(28.9)	235(32.7)		
Total (*3df*) §			0.0002	
Haplotype groups, no. (%)				
[T; T]/[T; T]	294(34.3)	97(27.1)		
[T; T]/[-;-]	435(50.8)	171(47.6)		
[-;-]/[-;-]	128(14.9)	91(25.3)	0.0004	1.53(1.23 ~ 1.96)

Notes: §For haplotype, *P* values were given by 2 × 4 table chi-square; ‘[-;-]’ means any other haplotypes; *OR* = odds ratio; *CI* = confidence interval; the association was analyzed by logistic regression analysis with adjustment for covariates, including age, sex, and alcohol consumption. *AsC*, asymptomatic carriers;*HBV*- *ALF*, patients with *HBV*-related acute liver failure.

## Discussion

In our case–control association study, we observed an increased risk of HBV-ALF in patients who carry *ESR1* c.30 C, c.453-397 C allele and [c.30 C; c.453-397 C] haplotype. This risk estimates did not change after adjustment for gender, age and alcohol consumption status, indicated that *ESR1* c.30 C, c.453-397 C allele and [c.30 C; c.453-397 C] haplotype is an independent risk factor. Combined with the novel evidences that the c.453-397 T > C polymorphism is a novel c.453-397 C allele-specific and c-myb-dependent enhancer-like cis-acting regulatory variation [20], our study emphasizes the importance of ESR1 in the pathophysiology of HBV-ALF on the population level. To our knowledge, this is the first report that the functional polymorphisms of *ESR1*gene may determine an individual’s susceptibility to HBV-ALF.

The design and results of our study manifest the features that are considered desirable components of an ideal association study [27]. These characteristics include a large cohort size, rigorous phenotypic selection, independent sample validation, plausible biological context, low *P* values, and appropriate statistical and LD test. Most HBV carriers are infected by maternal-infantile transmission in China. Both ALF cases and controls are HBV carriers in this study, which ensure all the subjects in the study having the history of HBV exposure, the association of *ESR1* polymorphisms with ALF may not due to population bias. To decrease the bias of age and sex on the effect of the estimate, we conducted stratification analysis for age and sex. Interestingly the association between the two *ESR1* polymorphisms (c.453-397 T > C and c.30 T > C) and HBV-ALF remained significant in both sex and patients at any age (with an age ≥ 40 years or < 40 years). The P value for the whole case control comparison is at the 10^-4^ level. According to the Better Associations for Disease and Genes (BADGE) system for describing genetic associations proposed by Manly [28] and the criteria proposed by Wacholder et al. [29], the false positive report probability (FPRP) of our results is very low. Although the highly significant association between *ESR1* polymorphisms and susceptibility to HBV-related ALF derives from biologically based *a priori* hypothesis, our initial findings should be independently verified in populations of different ancestry, especially in other East Asia populations, where HBV infection is the major cause of ALF. Furthermore, it is reasonable to speculate that the effects of the two SNPs on the occurrence of ALF may be different between postmenopausal and premenopausal women. Then, it will be better to conduct a stratification analysis for menopausal status in total HBV-ALF women. However, the number of potential postmenopausal women (N = 27) and premenopausal women (N = 34) in the HBV-ALF group is too small to gain enough statistical. In the following study, the expanded numbers of women patients with HBV-ALF will be help clarify the effects of the two SNPs on the occurrence of ALF between postmenopausal and premenopausal women.

It is conceivable that the common sequence variations in the *ESR1* gene affect the risk of different disease phenotype, such as breast cancer [30,31], osteoarthritis [32], cardiovascular disease [33], and migraine [34]. Our study group also has found that the *ESR1* polymorphisms influenced the susceptibility to persistent HBV infection [19], HBV-related liver cirrhosis [20] and hepatocellular carcinoma [21] and showed that the c.453-397 T > C polymorphism is a novel c.453-397 C allele-specific and c-myb-dependent enhancer-like cis-acting regulatory variation and could be part of the genetic variations underlying the susceptibility of individuals to HBV-related diseases [20]. The consistency of functional consequences is an additional strong point of our genetic epidemiological investigation.

Estrogen and variant ESR1 participates in the pathogenesis of HBV-related liver diseases [17,35]. During the course of chronic hepatitis B progressing to end-stage liver disease, variant forms of ESR1 predominates and sometimes becomes the only form expressed [17]. This variant lacks the estrogen-binding domain, but the intact DNA-binding domain maintains constitutive transcriptional activity, plays an important roles in the modulation of cell proliferation and inflammatory processes [36,37]. HBV carriers presenting variant ESR1 have an extremely aggressive clinical course.[13,17] Since variant ESR1 isoform predominates and sometimes becomes the only form expressed in the liver with endstage liver diseases, variant forms of ESR1 may be one of the possible mechanisms underlying the fact that c.453-397 T > C genetic variation influences susceptibility to HBV-ALF. our results, that the disease susceptible *ESR1* c.453-397 C allele had much stronger transcription activity than c.453-397 T allele, might suggest the liver failure risked c.453-397 C allele amplifies *ESR1* transcription and probably produces more variant ESR1 isoforms compared with c.453-397 T allele. Then patients carrying c.453-397 C allele secret more variant ESR1 and this elevated level of variant ESR1 reduces the response to the action of estrogen, and then risks to HBV-ALF. However, further studies are needed to identify the robust relationship between higher level of variant ESR1 secretion and c.453-397 T > C variation *in vivo*.

## Conclusions

In conclusion, our genetic epidemiological investigation suggests that the c.453-397 T > C polymorphism is related to the susceptibility to HBV-related liver failure and could be part of the genetic variations which underlies the phenotypic variation observed in individuals’ susceptibility to HBV-ALF in Chinese population. However, our conclusion need be supported by data from other populations. It is also interesting to investigate whether the *ESR1* intron region polymorphisms are associated with alcohol and drug induced liver failure in the west countries, where alcohol consumption and drug use are more popular.

## Competing interests

The funding sources had no role in study design, collection, analysis, or interpretation of data, or the writing of the report; or the decision to submit the report for publication. We declare that we have no conflict of interest to disclose.

## Authors’ contributions

Study design and discussion: ZY, GD, YW; sample collection: ZY, WT, YD, CD; data acquisition: ZY, WT, YD, WZ, CD; technical support: YD; data analysis and manuscript preparation: ZY, GD, WT; funding: ZY, GD; study supervision: YW, GD. All authors read and approved the final manuscript.

## Pre-publication history

The pre-publication history for this paper can be accessed here:

http://www.biomedcentral.com/1471-2350/13/49/prepub
